# Fragment-based determination of a proteinase K structure from MicroED data using *ARCIMBOLDO_SHREDDER*

**DOI:** 10.1107/S2059798320008049

**Published:** 2020-07-27

**Authors:** Logan S. Richards, Claudia Millán, Jennifer Miao, Michael W. Martynowycz, Michael R. Sawaya, Tamir Gonen, Rafael J. Borges, Isabel Usón, Jose A. Rodriguez

**Affiliations:** a Department of Chemistry and Biochemistry; UCLA–DOE Institute for Genomics and Proteomics; STROBE, NSF Science and Technology Center, University of California Los Angeles (UCLA), Los Angeles, CA 90095, USA; b Crystallographic Methods, Institute of Molecular Biology of Barcelona (IBMB–CSIC), Barcelona Science Park, Helix Building, Baldiri Reixac 15, 08028 Barcelona, Spain; c Howard Hughes Medical Institute, University of California Los Angeles (UCLA), Los Angeles, California, USA; d Department of Biological Chemistry, University of California Los Angeles (UCLA), Los Angeles, CA 90095, USA; e Department of Physiology, University of California Los Angeles (UCLA), Los Angeles, California, USA; f ICREA, Institució Catalana de Recerca i Estudis Avançats, Passeig Lluís Companys 23, 08003 Barcelona, Spain

**Keywords:** phasing, crystal, fragment, electron diffraction, cryoEM, MicroED, proteinase K, *ARCIMBOLDO_SHREDDER*

## Abstract

Structure determination of novel biological macromolecules by X-ray crystallo­graphy can be facilitated by the use of small structural fragments, some of only a few residues in length, as effective search models for molecular replacement to overcome the phase problem. Independence from the need for a complete pre-existing model with sequence similarity to the crystallized molecule is the primary appeal of *ARCIMBOLDO*, a suite of programs which employs this *ab initio* algorithm for phase determination. Here, the use of *ARCIMBOLDO* is investigated to overcome the phase problem with the electron cryomicroscopy (cryoEM) method known as microcrystal electron diffraction (MicroED). The results support the use of the *ARCIMBOLDO_SHREDDER* pipeline to provide phasing solutions for a structure of proteinase K from 1.6 Å resolution data using model fragments derived from the structures of proteins sharing a sequence identity of as low as 20%. *ARCIMBOLDO_SHREDDER* identified the most accurate polyalanine fragments from a set of distantly related sequence homologues. Alternatively, such templates were extracted in spherical volumes and given internal degrees of freedom to refine towards the target structure. Both modes relied on the rotation function in *Phaser* to identify or refine fragment models and its translation function to place them. Model completion from the placed fragments proceeded through phase combination of partial solutions and/or density modification and main-chain autotracing using *SHELXE*. The combined set of fragments was sufficient to arrive at a solution that resembled that determined by conventional molecular replacement using the known target structure as a search model. This approach obviates the need for a single, complete and highly accurate search model when phasing MicroED data, and permits the evaluation of large fragment libraries for this purpose.

## Abstract 2

A 1.6 Å resolution MicroED data set of proteinase K is phased using fragments derived from distantly related sequence homologues. *ARCIMBOLDO_SHREDDER* expands the phasing options for MicroED applications, overcoming the need for complete and highly accurate search models.

## 1. Introduction   

Crystallography has remained an indispensable method for structure determination since its initial demonstration over a century ago (Bragg & Bragg, 1913 ▸). Beyond X-ray diffraction, neutron and electron diffraction have contributed important advances to the crystallographic determination of macromolecular structures (Glaeser, 1999 ▸; Shi *et al.*, 2013 ▸; Gemmi *et al.*, 2019 ▸). Recently, an electron crystallography method called microcrystal electron diffraction (MicroED) has been developed to obtain high-resolution structures from frozen-hydrated three-dimensional macromolecular crystals (Supplementary Fig. S1; Nannenga, Shi, Hattne *et al.*, 2014 ▸). In MicroED, crystals of a few hundred nanometres in thickness are continuously rotated in an electron beam while diffraction is measured from a region of the specimen defined by the selected area aperture; the latter is positioned at the conjugate image plane of the objective lens. The recorded diffraction is reduced using conventional X-ray crystallography software to yield data that are suitable for structure determination. Phasing of MicroED data for biomolecules has been achieved by three approaches: by molecular replacement (Shi *et al.*, 2013 ▸), by direct methods (Sawaya *et al.*, 2016 ▸) or by using radiation damage (Martynowycz *et al.*, 2020 ▸). Refinement proceeds through programs such as *REFMAC* (Kovalevskiy *et al.*, 2018 ▸), *phenix.refine* (Afonine *et al.*, 2012 ▸) or *SHELXL* (Sheldrick, 2015*b* ▸) using electron scattering factors.

Important differences between X-ray and electron diffraction can impact the phase problem. Differences in wavelength (λ) impact Ewald sphere curvature such that λ is ∼1 Å in a typical X-ray experiment but ∼0.025 Å in a MicroED experiment performed using 200 keV electrons (Rodriguez & Gonen, 2016 ▸). While the relatively large scattering cross section of electrons in the 200–300 keV energy range is beneficial for extracting signal from very thin nanocrystals, the strong interaction between electrons and matter gives rise to a greater fraction of multiple scattering and absorption for thicker samples (Jansen *et al.*, 1998 ▸). These effects can be mitigated by using higher energy electrons, since penetration depth and kinematic scattering increase with electron beam energy, allowing thicker crystals to be interrogated at higher energies (300 keV). However, high beam energies increase the likelihood of knock-on radiation damage (Subramanian *et al.*, 2015 ▸). These challenges, combined with limited rotation of the electron microscope stage (∼140° maximally) and the possibility of low symmetry and some crystallites oriented preferentially on the grid surface, can lead to reduced completeness in MicroED movies from single crystals (Rodriguez & Gonen, 2016 ▸; Wennmacher *et al.*, 2019 ▸). Near-total completeness data sets are achieved through the merging of data from several crystals, but the merged data quality can be hindered by non-isomorphism as well as variations in crystal size and thickness at the nanoscale, all of which introduce difficulties in scaling (de la Cruz *et al.*, 2017 ▸). These, in addition to differences in X-ray and electron scattering factors (Colliex *et al.*, 2006 ▸), and limitations in the existing electron scattering factor libraries, make experimental phasing more challenging for electron diffraction applications, even without accounting for the impact of charged atoms (Yonekura *et al.*, 2015 ▸).

The phase problem is a common obstacle in all crystallo­graphic methods, including MicroED (Hattne *et al.*, 2015 ▸; Shi *et al.*, 2016 ▸). Determination of the first protein structure by MicroED, a 2.9 Å resolution structure of hen egg-white lysozyme, was achieved by molecular replacement using a known lysozyme polyalanine model (Shi *et al.*, 2013 ▸), akin to previous efforts in electron diffraction (Gonen *et al.*, 2005 ▸). No globular protein structure has been determined by direct methods from MicroED data; the approach has thus far only succeeded for MicroED of peptides and small molecules (Sawaya *et al.*, 2016 ▸; Genderen *et al.*, 2016 ▸). Isomorphous replacement methods have not yet been demonstrated for MicroED and this is considered to be a potentially intractable approach (Ceska & Henderson, 1990 ▸; Burmester & Schroeder, 1997 ▸). The lack of atomic absorption edges at the energies used for electron diffraction leaves little opportunity for anomalous dispersion-based phasing (Doyle & Turner, 1968 ▸; Burmester & Schroeder, 1997 ▸; Colliex *et al.*, 2006 ▸). Furthermore, initial efforts in structure determination by MicroED were overcast by concerns that dynamical scattering would scramble the intensities recorded from 3D protein crystals. The application of continuous rotation, yielding more accurate intensities (Nannenga, Shi, Leslie *et al.*, 2014 ▸), and the determination of novel biostructures has helped to dispel some of these concerns (see, for example, Rodriguez *et al.*, 2015 ▸).

A growing number of MicroED structures have been determined at resolutions outside the high-resolution regime by molecular replacement (Nannenga & Gonen, 2019 ▸). These include the structures of a fragment of α-synuclein at 1.4 Å resolution (Rodriguez *et al.*, 2015 ▸), of bovine liver catalase at 3.2 Å resolution (Nannenga, Shi, Hattne *et al.*, 2014 ▸) and of a Ca^2+^-ATPase at 3.2 Å resolution (Yonekura *et al.*, 2015 ▸). In each case, the use of near-ideal models also overcame potential issues with data quality that may pose barriers to phasing, including low completeness or high integration errors (Hattne *et al.*, 2015 ▸). With continued improvements to data collection and processing, novel structures continue to be determined by MicroED (Hughes *et al.*, 2018 ▸; Jones *et al.*, 2018 ▸; Purdy *et al.*, 2018 ▸; de la Cruz *et al.*, 2017 ▸; Zhou *et al.*, 2019 ▸; Xu *et al.*, 2018 ▸, 2019 ▸). Despite these successes, caution is prudent when evaluating the influence of model bias on the final structures, particularly where the model-to-structure r.m.s.d. is low; this has been the norm for many MicroED structures to date. Phasing in MicroED without atomic resolution data (∼1 Å) is a challenge and, given the complications regarding the experimental data, the phasing of protein structures by *ab initio* methods has immediate advantages: it does not require stereochemical knowledge, experimental modification of crystals or the collection of data at specific wavelengths (Hauptman, 1986 ▸; Sheldrick *et al.*, 2012 ▸; Usón & Sheldrick, 1999 ▸). Ultimately, atom placements must be computed whose transforms best correlate with the measured data and allow the generation of density maps that yield a refined structure (Sheldrick, 2015*a* ▸).

*ARCIMBOLDO* is a suite of software distributed within *CCP*4 (Winn *et al.*, 2011 ▸) that uses libraries of secondary-structure and tertiary-structure elements as initial search fragments for molecular replacement executed by *Phaser*, in which each fragment is oriented and positioned in the unit cell (McCoy *et al.*, 2007 ▸). Initial maps are then computed and improved by density modification using *SHELXE* (Thorn & Sheldrick, 2013 ▸). Finally, main-chain autotracing (Sheldrick, 2010 ▸) is performed to provide a reliable figure of merit at a given resolution in the form of a correlation coefficient (CC; Fujinaga & Read, 1987 ▸). In this way, *ARCIMBOLDO* substitutes the atomicity requirement in direct methods with the enforcement of secondary structure in order to accomplish fragment-based molecular replacement at resolutions near 2.0 Å (Rodríguez *et al.*, 2009 ▸).

*ARCIMBOLDO* can generate libraries of secondary-structure or tertiary-structure fragment search models in multiple ways (Rodríguez *et al.*, 2012 ▸; Medina *et al.*, 2020 ▸). The most effective search model in *ARCIMBOLDO* is an α-helix owing to its ubiquitous presence in protein structures, its constant geometry and its generally low *B* factors given its structural rigidity (Millán, Sammito & Usón, 2015 ▸). Libraries of idealized polyalanine helices can be generated for use in *ARCIMBOLDO_LITE* (Sammito *et al.*, 2015 ▸), while both secondary-structure and tertiary-structure elements can be made by extraction from the wide variety of existing structures deposited in the PDB using *ARCIMBOLDO_BORGES* (Sammito *et al.*, 2013 ▸). Another variant of *ARCIMBOLDO*, *ARCIMBOLDO_SHREDDER*, uses distantly related homologous structures to generate these polyalanine fragments for use as initial search models (Sammito *et al.*, 2014 ▸). This approach to molecular replacement eliminates the need for a single model and instead generates many possible models from low sequence-similarity homologues, low-resolution NMR structures or DNA-binding motifs (Pröpper *et al.*, 2014 ▸).

We now expand the available methods for the phasing of MicroED data at resolutions outside the atomic regime. We rely on fragments of homologue structures with low identity to the target for phasing using *ARCIMBOLDO_SHREDDER*. The success of this approach is demonstrated for proteinase K using a library of secondary-structure and tertiary-structure fragments from a distantly related homologous protein as molecular-replacement search models. An ensemble of polyalanine fragments from this library are placed and refined with density modification and autotracing in *SHELXE* (Usón & Sheldrick, 2018 ▸), obviating the need for a single closely related model to phase MicroED data.

## 2. Materials and methods   

### 2.1. Collection and processing of MicroED data for proteinase K   

The MicroED data sets originally used to produce the structures with PDB codes 5k7s (de la Cruz *et al.*, 2017 ▸) and 6cl7 (Hattne *et al.*, 2018 ▸) were integrated using *MOSFLM*. However, this procedure initially generated swayed intensities, as shown by analyzing normalized structure factors. This in turn suggested that the data might suffer from twinning when examined using an *L*-test. To address these issues, the data were reprocessed: they were indexed, integrated and scaled in *DIALS* and *XSCALE* to take advantage of 3D profile fitting (Clabbers *et al.*, 2018 ▸; Kabsch, 2010 ▸). Up to six high-damage frames were omitted from the end of each data set until no further increase in CC_1/2_ (Karplus & Diederichs, 2012 ▸) could be achieved to 1.6 Å resolution. Exhaustive merging was attempted for 12 data sets. The merging results were evaluated based on the resultant completeness and 〈*I*/σ(*I*)〉. The selection criterion was such that the merged data had >90% completeness using the fewest crystals and the highest 〈*I*/σ(*I*)〉. The final merged data set was from six crystals integrated to 1.6 Å resolution with an overall completeness of 91.5% and an 〈*I*/σ(*I*)〉 of 3.3 (Table 1 ▸; Supplementary Table S1).

### 2.2. *ARCIMBOLDO_SHREDDER* in sequential mode   

To accomplish fragment-based phasing from a homologous structure using *ARCIMBOLDO_SHREDDER*, a user-chosen homologue is first run through the *Phaser* rotation function. Positions from the peaks in the rotation search are clustered geometrically within a tolerance of 15° and each cluster is then used to systematically omit or extract segments from the template model. These segments are contiguous polyalanine fragments of variable length (Sammito *et al.*, 2014 ▸). The log-likelihood gain (LLG) scores of these fragments are then optimized by rotational analysis in *Phaser* (Storoni *et al.*, 2004 ▸). By comparing the LLG distribution for each sequentially ordered group of models of equal size, a single descriptor function, called the Shred-LLG function, is generated. Each point on the Shred-LLG function corresponds to a single residue and describes its individual contribution to the LLG score (Sammito *et al.*, 2014 ▸). Using this descriptor function, *ARCIMBOLDO_SHREDDER* then generates up to four models per rotation cluster. These are chosen by selecting peaks, plateaus, residues characterized by values above 75% of the maximum and values above the minimum peak height of the Shred-LLG function. These models are then run through independent *ARCIMBOLDO_LITE* searches, comprising both the *Phaser* location and refinement steps (McCoy *et al.*, 2005 ▸), and further trimming based on CC scores and successive rounds of density modification and main-chain auto­tracing with *SHELXE* until a final solution is reached (Supplementary Fig. S2). The parameters used for these *ARCIMBOLDO_SHREDDER* runs can be found in the supporting information (Section S1). These parameters, including the *SHELXE* inputs and fragment-generation options, can be varied for individual data sets, where data extrapolation can address a lack of completeness (Usón *et al.*, 2007 ▸). To inspire the best choice of these parameters, a tutorial describing our procedure for proteinase K structure determination by *ARCIMBOLDO* is available at http://chango.ibmb.csic.es/tutorial_microed.

### 2.3. *ARCIMBOLDO_SHREDDER* in spherical mode   

In spherical mode (Millán *et al.*, 2018 ▸), *ARCIMBOLDO_SHREDDER* produces a set of compact, overlapping models starting from a distant homologue template that are run as a library in *ARCIMBOLDO_BORGES*. To increase the radius of convergence of this approach, additional degrees of freedom are given to the models, which are decomposed in rigid-body groups and subjected to refinement against the intensity-based likelihood rotation-function target (Read & McCoy, 2016 ▸) and again after they have been placed in the unit cell. This refinement is accomplished in *Phaser* with the *gyre* and *gimble* modes (McCoy *et al.*, 2018 ▸), although other modifications of the model relying on the experimental data can be performed, such as normal-mode deformation (McCoy *et al.*, 2013 ▸) or pruning to optimize the CC (Sheldrick & Gould, 1995 ▸) or LLG (Oeffner *et al.*, 2018 ▸). Combination of partial solutions representing parts of a general hypothesis for the target fold can be performed in reciprocal space with *ALIXE* (Millán, Sammito, Garcia-Ferrer *et al.*, 2015 ▸). A functional set of parameters used for *ARCIMBOLDO_SHREDDER* in a spherical mode run can be found in the supporting information (Section S2).

### 2.4. Homologues used as models for *ARCIMBOLDO_SHREDDER* and for molecular replacement   

Using the sequence corresponding to the proteinase K structure deposited as PDB entry 5k7s, we searched for homologous structures using the *HHpred* server (Söding *et al.*, 2005 ▸). Model libraries were generated from structures with PDB codes 4dzt (B. L. Barnett, P. R. Green, L. C. Strickland, J. D. Oliver, T. Rydel & J. F. Sullivan, unpublished work), 5yl7 (Park *et al.*, 2018 ▸), 5jxg (Dahms *et al.*, 2016 ▸) and 1ga6 (Wlodawer *et al.*, 2001 ▸). Using *GESAMT* (Krissinel, 2012 ▸), the r.m.s.d. values for the models with PDB codes 4dzt, 5yl7, 5jxg and 1ga6 were 1.01, 1.43, 1.87 and 2.10 Å based on the alignment of 268, 245, 247 and 226 residues, respectively (Supplementary Table S2). Libraries of between 100 and 200 models were generated from these structures by *ARCIMBOLDO_SHREDDER* in spherical or sequential mode and were evaluated using *ARCIMBOLDO_BORGES*.

### 2.5. Refinement and analysis of the proteinase K structure determined by *ARCIMBOLDO_SHREDDER* in sequential mode with PDB entry 4dzt   

An initial *ARCIMBOLDO_SHREDDER* solution determined from a set of 13 traced segments derived from the model with PDB code 4dzt and totaling 175 residues was refined in *Phenix* using *phenix.refine* (Liebschner *et al.*, 2019 ▸; Afonine *et al.*, 2012 ▸). Subsequent visualization and model building were performed in *Coot* (Emsley *et al.*, 2010 ▸). Fragment chains were first connected by building along the full length of the protein backbone. Side chains were then assigned unambiguously, and finally ordered waters were placed. The reported r.m.s.d. values were computed by secondary-structure matching superposition (Krissinel & Henrick, 2004 ▸) using *Super* in *PyMOL* (version 1.8; Schrödinger) or *GESAMT* (Krissinel, 2012 ▸), considering only core C^α^ atoms. Placed fragments were evaluated against our final, fully refined model by calculating the LLG with *Phaser* and the initial CC and weighted mean phase error (wMPE) with *SHELXE*.

## 3. Results   

### 3.1. Using *ARCIMBOLDO_SHREDDER* in sequential mode to phase proteinase K   

**Figure 1:**
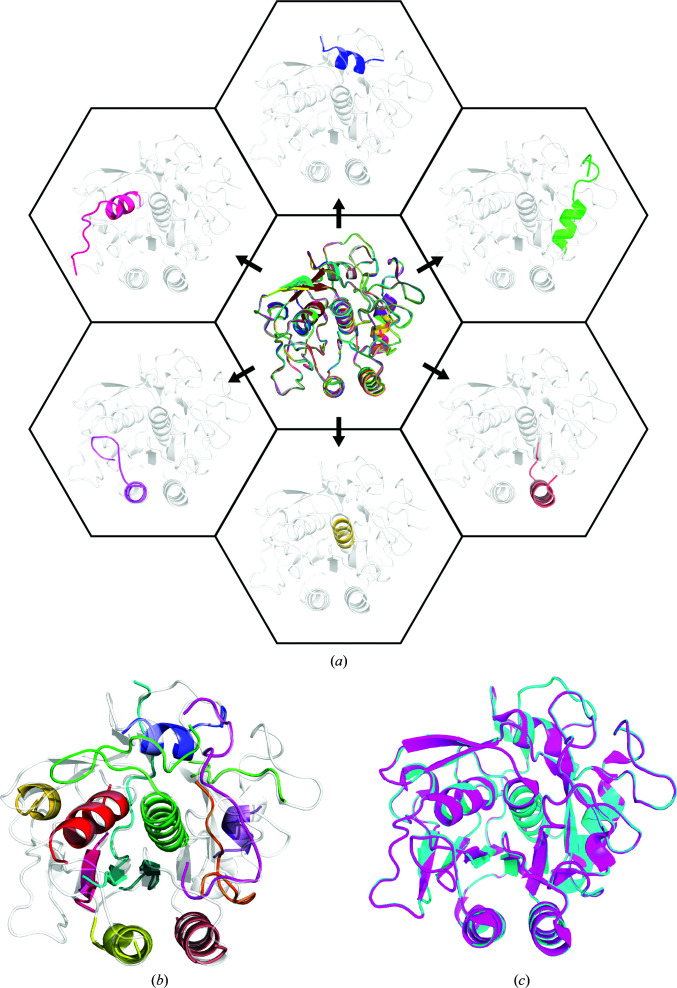
Schematic of fragment generation and structure determination of proteinase K using *ARCIMBOLDO_SHREDDER*. (*a*) At the center, an overlay of all 759 fragments sequentially generated from the template model (PDB entry 4dzt) is shown. Examples of individual fragments derived from the model template are shown extracted out of the center model in the context of the final structure of proteinase K. (*b*) The output solution from *ARCIMBOLDO_SHREDDER*, composed of the 13 placed individual fragments (colored chains), is shown overlaid with the final structure of proteinase K. (*c*) The final structure of proteinase K determined with *ARCIMBOLDO_SHREDDER* (PDB entry 6v8r; cyan) overlaid with the previously determined MicroED structure (PDB entry 5k7s; pink) gives a C^α^ r.m.s.d. of 0.12 Å.

*ARCIMBOLDO_SHREDDER* was implemented to phase MicroED data using a 1.6 Å resolution data set for proteinase K that was collected as described previously (Hattne *et al.*, 2018 ▸) and used in part in the determination of the structures deposited as PDB entries 5k7s and 6cl7. This data set (Table 1 ▸), which is 91.49% complete to 1.6 Å resolution with an overall 〈*I*/σ(*I*)〉 of 3.3, was suitable for molecular replacement using a known crystal structure of proteinase K (Table 1 ▸). To evaluate fragment-based phasing, we chose a homologue of proteinase K with a sequence identity of 40% (PDB entry 4dzt). This structure has a 268-atom C^α^ r.m.s.d. of 1.01 Å to the proteinase K structure determined from these data, as calculated by *GESAMT*. A library of models was generated from this starting template based on rotational analysis by *Phaser* using electron scattering factors, with top-scoring clusters of rotation solutions being used to perform an analysis of the effect of omitting continuous spans of the structure. Such omit fragments were generated by extracting 10–20-residue contiguous segments every four residues for the length of the protein, resulting in a total of 759 polyalanine fragments (Fig. 1 ▸ *a*). The global evaluation of such fragments is performed in terms of a Shred-LLG function, which through joint scoring of the results obtained using all of these fragments assesses the local accuracy of the initial template (described in Section 2). The located models were input to *SHELXE* for autotracing expansion as outlined above. This implementation resulted in a correct output model composed of 175 residues encompassing 13 chains obtained from a solution characterized by a *Phaser* rotation LLG score of 287.30, a *Phaser* translation *Z*-score (TFZ) of 20.40 and a *SHELXE* final CC of 23.31% (Fig. 1 ▸ *b*).

**Figure 2:**
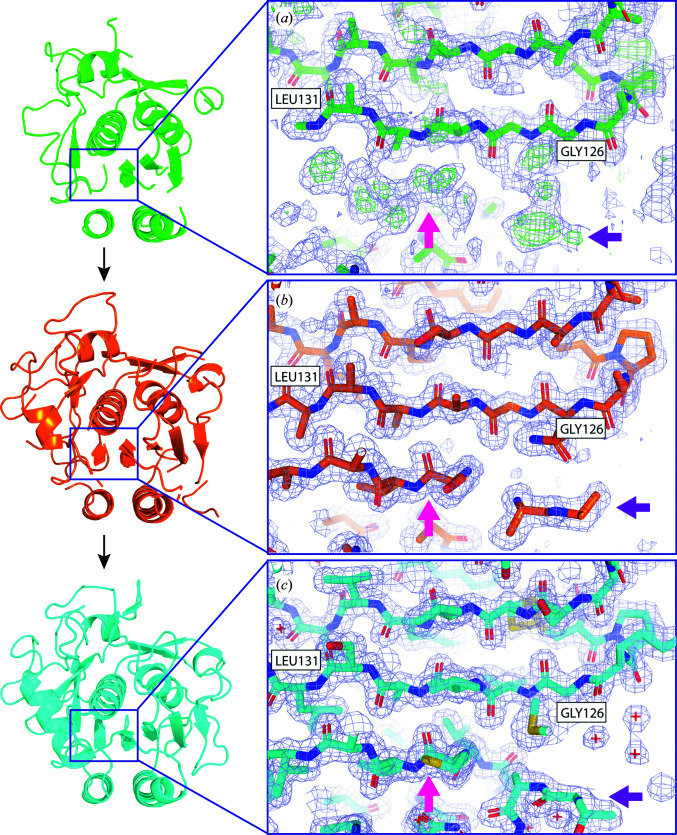
Building of missing structural elements. Starting from an *ARCIMBOLDO*-generated solution, advancement in refinement is shown in stages including (*a*) the initial *ARCIMBOLDO* output, (*b*) an intermediate stage of building and (*c*) the final structure (PDB entry 6v8r). Pink and purple arrows indicate positions in the map where structural elements, a β-sheet (pink) and a loop region (purple), were built into the positive difference-map peak density seen in the initial map.

**Figure 3:**
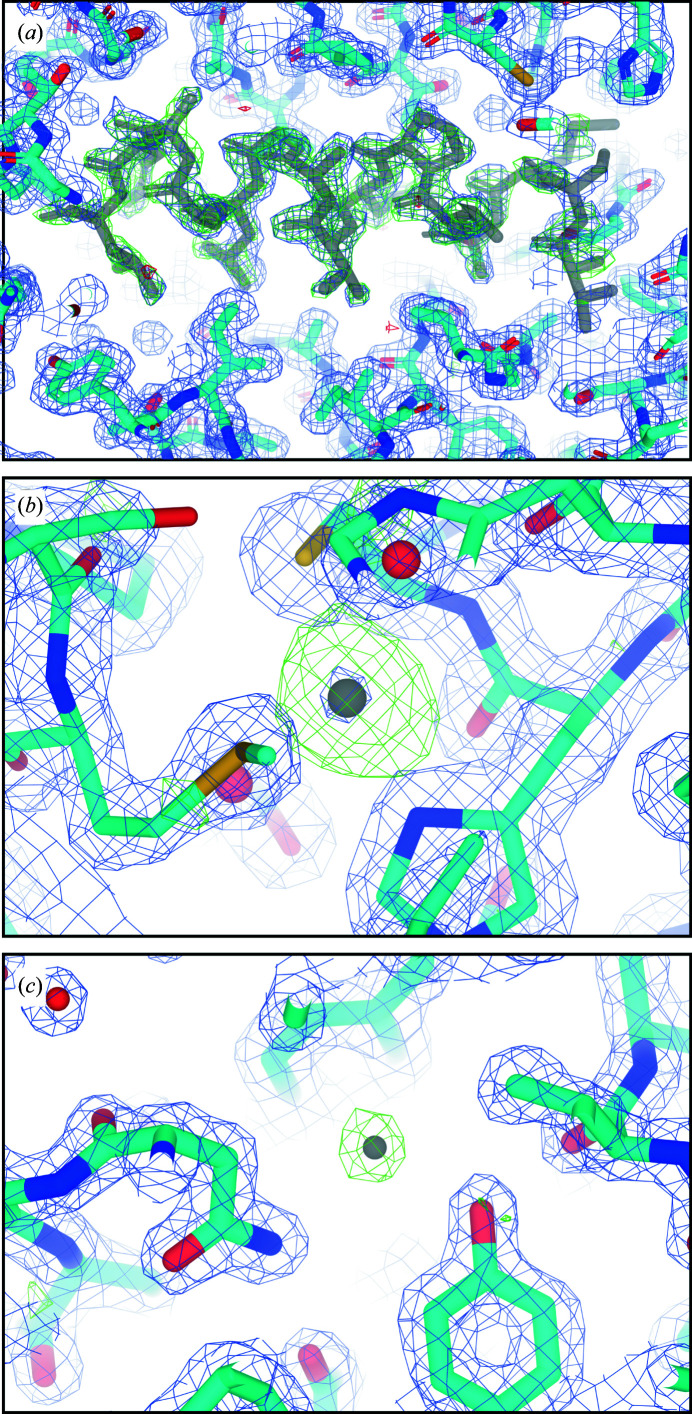
Representative omit maps. (*a*) Omit map for the *ARCIMBOLDO_SHREDDER* solution generated after removal of the sixth α-helix comprised of residues 223–237 (shown in gray). (*b*) Omit map generated after the removal of one of the calcium ions coordinated by the structure (shown in gray). (*c*) Omit map generated after the removal of one representative water molecule (shown in gray). The blue mesh is the 2*mF*_o_ − *F*_c_ map contoured at 1.5σ and the green mesh is the *F*_o_ − *F*_c_ map contoured at 3.0σ.

The output model traced by *SHELXE* was composed of fragments from seven α-helices and three β-strands as well as a few loop regions that are conserved between proteinase K and the homologue. Missing structural elements appeared clearly as positive difference-map peaks in initial refinements (Fig. 2 ▸ *a*), and subsequent rounds of manual model building and refinement revealed missing loops, side chains and ordered waters (Figs. 2 ▸ *b* and 2 ▸ *c*). The refined structure solution contained 279 unambiguously assigned residues and 122 ordered waters, and had a final *R*_work_ of 19.6% and *R*_free_ of 23.3%. Omit maps computed from the refined *ARCIMBOLDO_SHREDDER* solution or the solution determined by *Phaser* using PDB entry 4dzt as a search model, having deleted from each the sixth helix corresponding to residues 223–237 in proteinase K, resulted in positive difference density that outlined not only the location of the helix, but also revealed a continuous map at 3.0σ matching the appropriate side chains for all but four residues in the helix (Fig. 3 ▸ *a*). Additionally, omit maps of the two coordinated calcium ions give positive difference-map peaks at 17.69σ and 11.73σ (Fig. 3 ▸ *b*), and the omit map for the removal of an ordered water molecule gives rise to a 6.95σ positive-density peak (Fig. 3 ▸ *c*). Placement of bound ions and waters satisfied the difference map density and resulted in a decrease in the *R* factors.

### 3.2. Comparison of the solution from *ARCIMBOLDO_SHREDDER* with the known proteinase K structure   

The 13 homologue fragments placed by *Phaser* overlay well with the final structure of proteinase K (Fig. 1 ▸ *b*). The structure determined using model fragments from this *ARCIMBOLDO_SHREDDER* run is nearly identical to the previously determined MicroED structure of proteinase K (Hattne *et al.*, 2018 ▸), with a C^α^ r.m.s.d. of 0.12 Å (Fig. 1 ▸ *c*). The input model aligned with the known structure of proteinase K gives a C^α^ r.m.s.d. of 0.65 Å when aligning 232 atoms and yields a correct solution when used for molecular replacement. Notably, the *Phaser* LLG and TFZ scores are lower for this solution (179 and 19.6, respectively) compared with the initial scores for the *ARCIMBOLDO_SHREDDER* solution, showing that the fragments placed reflect accurate structural components that are present in the final structure.

### 3.3. Use of spherical fragment generation for structure determination   

To further evaluate the potential of model improvement against the experimental MicroED data, we also attempted phasing using the recently developed spherical mode in *ARCIMBOLDO_SHREDDER* (Millán *et al.*, 2018 ▸). This mode is particularly appropriate for more structurally distant homologs that have an overall conserved fold and where deviations from the final model are distributed isotropically in Cartesian space. In such a case, simply removing the regions of largest deviation or extruding contiguous fragments, as is performed in sequential mode, may not be sufficient to obtain a phasing solution. Instead, in spherical mode, small compact fragments of pre-defined size are extracted from the distant homologue, given degrees of freedom and searched for independently, and subsequently combined in reciprocal space (Millán *et al.*, 2020 ▸). The spherical mode in *ARCIMBOLDO_SHREDDER* selects the size of its models based on the eLLG score. Given the data resolution, the expected r.m.s.d. of the models and a target eLLG (by default 30), the appropriate size for the models is derived. All of the models produced in the run were within a range of ten residues of such a value. The models ranged in size between 44 and 48 residues. Three homologues with various degrees of sequence identity and structural similarity (PDB entries 5yl7, 5jxg and 1ga6), which did not produce viable solutions in sequential mode, were evaluated using *ARCIMBOLDO_SHREDDER* in spherical mode to attempt phasing of the proteinase K MicroED data. The results from this attempt are summarized in Table 2 ▸ and demonstrate the determination of correct partial solutions using this method. However, while solutions are identified during the search, the extension of these partial solutions in *SHELXE* can be notably more difficult for MicroED data than for X-ray data. This may be owing in part to the high initial mean phase errors (68–76°) associated with the placement of these fragments. This in turn will require improved algorithms, implemented in *SHELXE*, that take into account the unique aspects of electron scattering.

### 3.4. Comparing the performance of *ARCIMBOLDO_SHREDDER* in spherical mode against both MicroED and X-ray diffraction data using more distant homologues of proteinase K   

The same homologues used for phasing the MicroED data in the experiments described in Section 3.4 and Table 2 ▸ were used to phase an X-ray data set from an isostructural form of proteinase K: PDB entry 4woc (Guo *et al.*, 2015 ▸). Attempts at phasing using the X-ray data set are summarized in Table 3 ▸. With these data, fragment placement succeeds in generating correct placements with all models tested against both data sets. As expected, these trials yield better minimum wMPEs with X-ray data than with MicroED data. The overall trend in both cases favored the placement of fragments from structures with higher similarity to the known solution. For example, from the library of models generated by *ARCIMBOLDO_SHREDDER* from the proteinase K structure deposited as PDB entry 5yl7, 39 fragments were placed and yielded correct solutions in the MicroED data set. The best showed a weighted mean phase error (wMPE) of 68.8°. In the most extreme of cases, fragments generated from a pepstatin-insensitive carboxyl proteinase from *Pseudomonas* sp. 101 (PSCP) deposited as PDB entry 1ga6 (with only 21% sequence identity to the target) facilitated the placement of two correct fragments as solutions, with the best having a wMPE of 76°. These tests collectively demonstrate the promise of some distant homologues for the accurate placement of fragments using MicroED data.

### 3.5. Phasing with idealized helices as search models in *ARCIMBOLDO_LITE*   

Searches using idealized helix models ranging in size from three to 18 alanine residues were attempted on the MicroED data set (PDB entry 6v8r) and an example X-ray proteinase K data set (PDB entry 4woc). The parameters for these runs were set to the defaults for *ARCIMBOLDO_LITE* except for the implementation of electron scattering factors in *Phaser* for the 6v8r data set (supporting information, Section S3). None of these runs with either data set produced a solution with a wMPE of lower than 85°, indicating that no viable solution was identified by this method. This result is not surprising given that the helix fragments represent a very small scattering fraction of the full structure.

## 4. Discussion and conclusions   

As the field of MicroED continues to expand, a growing number of novel structures may present phasing hurdles. Given that experimental phasing remains a challenge in MicroED, it is important to explore other ways to overcome the phase problem beyond direct methods and molecular replacement. To date, more than a dozen *ab initio* structures determined by direct methods from MicroED data have been deposited in the PDB, in comparison to several dozen structures determined by conventional molecular replacement with resolutions between 1.2 and 3 Å (Rodriguez & Gonen, 2016 ▸). Of the set determined by molecular replacement, approximately 13 are in some way novel, although many of these rely on highly similar search models determined by X-ray diffraction. The relatively low number of completely novel structures is due in part to the challenges associated with the experimental phasing of MicroED data. Given the smaller difference in scattering between heavy and light atoms in electron diffraction compared with X-ray diffraction, experimental phasing by isomorphous replacement remains un­demonstrated and, at least for 2D crystals, might be intractable (Ceska & Henderson, 1990 ▸).

Many of the structures determined by MicroED to date have resolutions (1.2–2 Å) appropriate for attempts at phasing by *ARCIMBOLDO* or other fragment-based and *ab initio* phasing approaches. Fragment-based approaches are typically less restrictive than conventional molecular-replacement methods for phasing and have been demonstrated in electron crystallography of 2D and 3D crystals using image data combined with electron diffraction data (Wisedchaisri & Gonen, 2011 ▸). Requirements that are important for the success of structure determination by *ARCIMBOLDO* from both MicroED and X-ray diffraction data include (i) high completeness, (ii) data quality and perhaps resolution and (iii) models similar to the target structure from which fragments are derived. When these criteria are met, conventional molecular replacement is often also successful. For instance, phasing of polymeric amyloid peptide assemblies has been achieved using idealized β-strands that closely match the final geometry of the polypeptide structure (Rodriguez *et al.*, 2015 ▸).

Overall, while fragment placement succeeds with a variety of libraries, even those with distant homology to the known target structure for MicroED data, the extension of partial solutions remains a challenge. This may result in part from the nature of the maps, which represent a screened Coulomb potential rather than electron density, or from inherent features of the data. Additional limitations are likely to be present in MicroED maps. For example, some crystals may suffer from orientation bias on an EM grid, and this in turn may lead to a missing cone of information which can persist despite attempts at merging multiple data sets (Nannenga, Shi, Hattne *et al.*, 2014 ▸). Problems also arise from inaccuracies in the estimation of standard errors of the experimental data. The strong effects of anisotropy (Strong *et al.*, 2006 ▸) and the partial effects of directional lack of completeness, along with potential absorption and dynamic scattering (Cowley & Moodie, 1957 ▸; Dorset *et al.*, 1992 ▸; Glaeser & Downing, 1993 ▸), can add to a uniquely deleterious effect on maps and thus may influence density modification and autotracing. Despite these, density modification has been demonstrated for electron diffraction (Wisedchaisri & Gonen, 2011 ▸). The use of electron scattering form factors, data filtering by information content (Read *et al.*, 2020 ▸) and anisotropy correction are expected to be beneficial for these approaches, both during direct-method protocols and with fragment-based approaches. Future corrections implemented during data reduction may ameliorate these effects. Our present observations suggest that *ARCIMBOLDO* may be successful in identifying phasing solutions for MicroED data from structures of distantly related homologues. Various modes of search-model definition, be it linear fragments, structures with omitted segments or spherical regions of structures, could yield solutions with varying success.

After years of successful application to X-ray crystallo­graphic data, this study demonstrates the utility of fragment-based phasing methods and *ARCIMBOLDO* with MicroED data. Our ability to determine a known structure using small structural fragments derived from a distantly related homologue opens the possibility of the *de novo* determination of structures by MicroED. This demonstration follows several reports of fragment-based phasing or phase extension for electron diffraction data (Wisedchaisri & Gonen, 2011 ▸). Phasing methods that employ the use of fragments are gaining in popularity for the determination of X-ray structures. An example of these is *AMPLE* (Bibby *et al.*, 2012 ▸; Rigden *et al.*, 2018 ▸), which in turn uses *ROSETTA* (Qian *et al.*, 2007 ▸), *QUARK* (Keegan *et al.*, 2015 ▸) or *CONCOORD* (de Groot *et al.*, 1997 ▸) to generate models. Some of these programs offer the possibility of generating *ab initio* fragments derived from the target sequence, for example *FRAGON* (Jenkins, 2018 ▸) and *FRAP* (Shrestha & Zhang, 2015 ▸). While the limited substrate scope of our study precludes conclusions on the general application of fragment-based phasing to MicroED data, our results demonstrate that fragment-based phasing is advantageous when applied to MicroED data with a resolution that is too poor for direct methods. In such cases, *ARCIMBOLDO_SHREDDER* and perhaps other fragment-based phasing programs offer a potential solution to a problem that may otherwise remain unsolved.

## 5. Related literature   

The following references are cited in the supporting information for this article: Arndt & Wonacott (1977 ▸), Nannenga & Gonen (2016 ▸).

## Supplementary Material

PDB reference: proteinase K, 6v8r

ARCIMBOLDO parameters and Supplementary Figures and Tables. DOI: 10.1107/S2059798320008049/rr5196sup1.pdf

## Figures and Tables

**Table 1:** MicroED structure of proteinase K determined by fragment-based phasing

Phasing method	*ARCIMBOLDO_SHREDDER* (fragment library from PDB entry 4dzt)	*Phaser* (PDB entry 4dzt)
Data collection and processing		
No. of crystals	6	6
Total electron exposure (e Å^−2^)	0.86	0.86
Molecular weight (kDa)	28.9	28.9
Resolution (Å)	55.79–1.60 (1.657–1.600)	55.79–1.60 (1.657–1.600)
Space group	*P*4_3_2_1_2	*P*4_3_2_1_2
*a*, *b*, *c* (Å)	67.25, 67.25, 99.92	67.25, 67.25, 99.92
α, β, γ (°)	90, 90, 90	90, 90, 90
Total No. of reflections	194052	194052
No. of unique reflections	29058 (2506)	29058 (2506)
CC_1/2_	0.912 (0.051)	0.912 (0.051)
〈*I*/σ(*I*)〉	3.31	3.31
Completeness (%)	91.49 (66.19)	91.49 (66.19)
Multiplicity	6.68	6.68
Phasing		
Residues placed	175	
Fragments placed	13	
LLG	287.3	179
TFZ	20.4	19.6
CC (%)	23.31	
Refinement		
Resolution (Å)	55.79–1.60 (1.657–1.600)	55.79–1.60 (1.657–1.600)
*R*_work_ (%)	19.6	19.8
*R*_free_ (%)	23.3	23.7
RSCC	0.92	0.92
No. of residues	279	279
No. of protein atoms	2056	2038
No. of water molecules	122	138
No. of ligand atoms	2	2
Average *B* factor (Å^2^)		
Overall	17.37	16.84
Protein	16.96	16.64
Water	18.89	19.72
Ligand	25.41	25.76
R.m.s.d., bonds (Å)	0.007	0.007
R.m.s.d., angles (°)	0.9	0.9
Ramachandran statistics		
Outliers (%)	0.36	0.36
Favored (%)	97.11	97.11
Clashscore	6.75	5.28

**Table 2:** Results for fragment-based phasing of the MicroED data set

Target: 5k7s	Identity†	R.m.s.d. (*GESAMT*) (Å)/No. of residues	No. of correct solutions/total solutions	Best wMPE (°)
5yl7	0.310	1.43/245	39/394	68.8
5jxg	0.193	1.87/247	6/375	72.4
1ga6	0.208	2.10/226	2/632	76.0

† Identity is denoted as a fraction, where 1 represents perfect identity.

**Table 3:** Results for fragment-based phasing of the X-ray data set associated with PDB entry 4woc

Target: 5k7s	Identity†	R.m.s.d. (*GESAMT*) (Å)/No. of residues	No. of correct solutions/total solutions	Best wMPE (°)
5yl7	0.310	1.43/245	25/579	64.5
5jxg	0.193	1.87/247	10/486	71.0
1ga6	0.208	2.10/226	4/595	76.5

† Identity is denoted as a fraction, where 1 represents perfect identity.
