# Supplementary material for: Fragment-based determination of a proteinase K structure from MicroED data using ARCIMBOLDO_SHREDDER
Source: Acta Crystallogr D Struct Biol. 2020 Jul 27;76(Pt 8):703–12. doi: 10.1107/S2059798320008049 (PMC7397493; doi:10.1107/S2059798320008049)
Supplement: Supplementary file 1 [file d-76-00703-sup1.pdf]

STRUCTURAL
BIOLOGY

Volume 76 (2020)

Supporting information for article:

Fragment-based determination of a proteinase K structure from MicroED data using *ARCIMBOLDO_SHREDDER*

Logan S. Richards, Claudia Millán, Jennifer Miao, Michael W. Martynowycz, Michael R. Sawaya, Tamir Gonen, Rafael J. Borges, Isabel Usón and Jose A. Rodriguez

S1. Parameters for running ARCIMBOLDO_SHREDDER in sequential mode

[CONNECTION]

distribute_computing = multiprocessing

[GENERAL]

working_directory =

mtz_path =

hkl_path =

ent_path =

[ARCIMBOLDO-SHREDDER]

formfactors: FORMFACTORS ELECTRON

shred_range: 10 20 4 fragment

name_job = output

molecular_weight = 28900

number_of_component = 1

i_label = IMEAN

sigi_label = SIGIMEAN

model_file =

rmsd_shredder = 1.2

trim_to_polyala = True

shred_method = sequential

shelxe_line = -m15 -a8 -s0.5 -v0 -t10 -q -o -y1.60

shelxe_line_last = -m15 -a1 -s0.45 -v0 -t10 -q -o -y1.60 -e1.10

[LOCAL]

path_local_phaser =

path_local_shelxe =

S2. Parameters for running ARCIMBOLDO_SHREDDER in spherical mode

[CONNECTION]:

distribute_computing: local_grid

setup_bor_path =

[GENERAL]

working_directory =

mtz_path =

hkl_path =

ent_path =

[ARCIMBOLDO-SHREDDER]

formfactors: FORMFACTORS ELECTRON

name_job =

molecular_weight = 28900

number_of_component = 1

i_label = IMEAN

sigi_label = SIGIMEAN

model_file =

rmsd_shredder = 0.8

savephs: True

alixe: False

shred_method = spherical

shelxe_line = -m15 -a8 -s0.45 -v0 -t6 -y1.60 -e1.10

shelxe_line_last = -m15 -a1 -s0.45 -v0 -t6 -y1.60 -e1.10

S3. Parameters for running ARCIMBOLDO_LITE

[CONNECTION]

distribute_computing = multiprocessing

[GENERAL]

pdb_path =

working_directory =

mtz_path =

hkl_path =

ent_path =

[ARCIMBOLDO]

formfactors = FORMFACTORS ELECTRON

rmsd = 0.8

i_label = IMEAN

sigi_label = SIGIMEAN

name_job =

molecular_weight =

helix_length = 12

[LOCAL]

path_local_phaser =

path_local_shelxe =

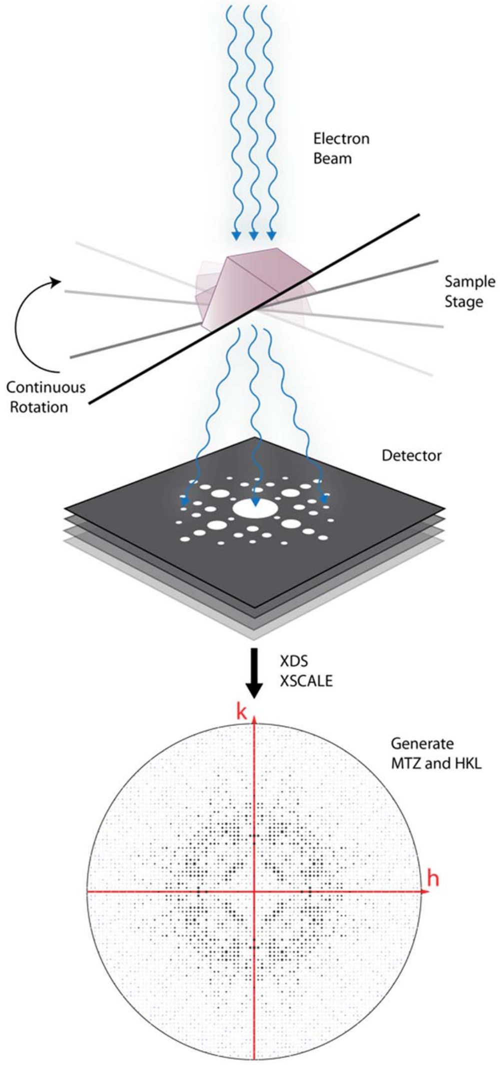

Figure S1 Schematic of continuous rotation MicroED data collection. An electron beam illuminates a protein crystal resting on a cryoEM grid. The microscope sample stage rotates continuously as the scattered electrons are detected below in a process analogous to the rotation method in macromolecular X-ray crystallography (Arndt & Wonacott, 1977; Nannenga *et al*., 2014). The recorded intensities are then processed to generate the MTZ and HKL data files for structure solution.

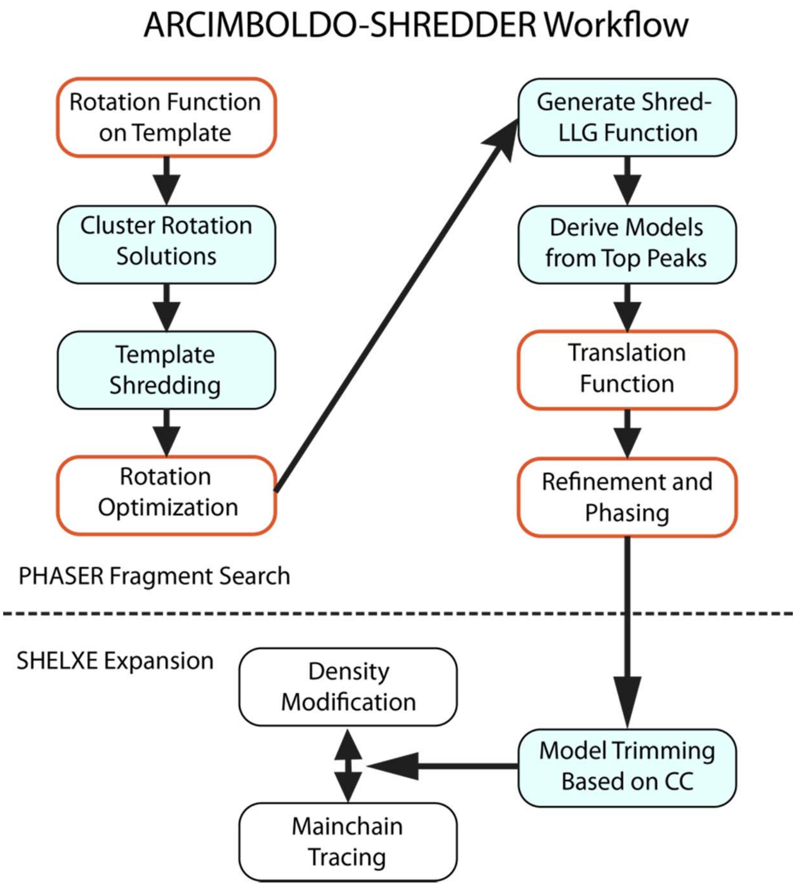

Figure S2 ARCIMBOLDO_SHREDDER workflow. A chosen template model undergoes rotational analysis in Phaser and top scoring peaks are clustered geometrically. These clusters are used to sequentially generate short fragments from the template which then individually undergo rotational analysis in Phaser, the results of which are used to define the Shred-LLG function. Based on the Shred-LLG function models are generated which combine top scoring fragments. These models undergo translational analysis, refinement, and phasing in Phaser. Finally, these solutions are sent into SHELXE where each model is further trimmed based on CC and is subjected to iterative cycles of density modification and mainchain auto-tracing until a final solution is achieved. ARCIMBOLDO operations are shown with a blue background and Phaser operations using electron scattering factors are shown outlined in red.

Table S1 Statistics of individual tilt series merged to obtain the 6V8R dataset

**Movie**	**1**	**5**	**6**	**8**	**9**	**10**
**Resolution (Å)**	55.79-1.6	55.79-1.6	55.79-1.6	55.79-1.6	55.79-1.6	55.79-1.6
**Completeness**	41.5%	34.0%	29.4%	32.6%	49.7%	29.6%
**CC1/2**	93.5	94.3	95.5	96.9	95.8	94.6
**<I/σI>**	1.93	2.06	2.81	3.10	2.47	2.14
**Multiplicity**	2.64	2.12	5.68	2.67	2.45	2.21
**R-factor**[*]	34.9%	23.5%	40.1%	18.3%	25.3%	26.6%

[*] As defined by XSCALE.

Table S2 Summary of HHpred results and structural alignments[*]

**PDB**	**Identity**	**Prob.**	**E-value**	**Align length**	**RMSD_SSM**	**RMSD_GESAMT**
5YL7	0.31	100	5.4e-30	344	1.7 Å / 245 res	1.43 Å / 245 res
5JXG	0.193	99.9	2.3e-29	332	2.03 Å /241 res	1.87 Å / 247 res
1GA6	0.208	99.9	6.4e-25	355	2.32 Å / 216 res	2.1 Å / 226 res

[*] Values obtained from HHpred reported as of March 2020.
